# 2D/3D CMR tissue tracking versus CMR tagging in the assessment of spontaneous T2DM rhesus monkeys with isolated diastolic dysfunction

**DOI:** 10.1186/s12880-018-0288-y

**Published:** 2018-11-26

**Authors:** Tong Zhu, Wen Zeng, Yushu Chen, Yu Zhang, Jiayu Sun, Zhigang Liang, Zunyuan Yang, Wei Cheng, Lei Wang, Bin Song, Bing Wu, Fangtong Wang, Yinan Liang, Li Gong, Jie Zheng, Fabao Gao

**Affiliations:** 1 Department of Radiology, West China Hospital, Sichuan University, 37 Guoxue alley, Chengdu, 610041 Sichuan Province China; 2 Sichuan Primed Shines Bio-tech Co., Ltd, Chengdu, China; 3 Mallinckrodt Institute of Radiology, Washington University School of Medicine, St. Louis, MO USA

**Keywords:** Diabetic cardiomyopathy, Rhesus monkey, Cardiac MRI, Tissue tracking, Tagging

## Abstract

**Background:**

Spontaneous T2DM in rhesus monkeys manifests as isolated diastolic dysfunction in the early stage of diabetic cardiomyopathy, similar to humans. Myocardial deformation measurements have emerged as a superior way to measure left ventricular (LV) function in the early stage of cardiac dysfunction, making it possible to further evaluate early-stage LV dysfunction in spontaneous T2DM rhesus monkeys.

**Methods:**

Spontaneous T2DM rhesus monkeys with isolated diastolic dysfunction (T2DM-DD, *n* = 10) and corresponding nondiabetic healthy animals (ND, *n* = 9) were prospectively scanned for a CMR study. Circumferential and longitudinal peak systolic strain (Ecc, Ell), time to peak strain (tEcc, tEll) and peak diastolic strain rate (CSR, LSR) obtained from 2D/3D CMR-TT were compared with those obtained from CMR tagging separately. In addition, all CMR imaging protocols were performed twice in 9 ND animals to assess test-retest reproducibility.

**Results:**

Compared with the ND group, the T2DM-DD monkeys demonstrated significantly impaired LV Ecc (− 10.63 ± 3.23 vs − 14.18 ± 3.19, *p* < 0.05), CSR (65.50 ± 14.48 vs 65.50 ± 14.48, *p* < 0.01), Ell (− 9.11 ± 2.59 vs − 14.17 ± 1.68, *p* < 0.05), and LSR (59.43 ± 19.17 vs 108.46 ± 22.33, p < 0.01) with the tagging. Only Ecc (− 13.10 ± 2.47 vs − 19.03 ± 3.69, p < 0.01) and CSR (148.90 ± 31.27 vs 202.00 ± 51.88, p < 0.01) were significantly reduced with 2D CMR-TT, and only Ecc (− 13.77 ± 1.98 vs − 17.26 ± 3.78, p < 0.05) was significantly reduced with 3D CMR-TT. Moreover, 2D/3D CMR-TT-derived Ecc and CSR correlated with the corresponding tagging values collectively, with a statistically significant ICC value (p < 0.05). Test-retest repeatability analysis showed that most tagging-derived biomarkers had acceptable repeatability (p < 0.01). In addition, 2D CMR-TT-derived indicators were poorer than those derived from the tagging method but better than those obtained using the 3D method, with larger ICCs except for tEcc (p < 0.05).

**Conclusions:**

LV systolic and diastolic deformations were impaired in spontaneous T2DM rhesus monkeys previously diagnosed with isolated diastolic dysfunction by echocardiography. The 2D CMR-TT-derived Ecc and CSR were effective in the evaluation of the myocardial systolic and diastolic functions of early-diabetic cardiomyopathy, with relatively higher test-retest reproducibility and acceptable correlation with the tagging method compared with the 3D CMR-TT method.

## Background

Diabetic cardiomyopathy (DCM) is a clinical condition of ventricular dysfunction that occurs in the absence of coronary atherosclerosis and hypertension in patients with diabetes mellitus [1]. It significantly increases the prevalence of heart failure and is associated with considerably worse clinical outcomes [2]. Sufficiently sensitive diagnostic approaches in the early stage of DCM and novel strategies to reduce the risk of heart failure in diabetes patients are needed [3].

Such approaches and strategies require the use of appropriate animals for preclinical and translational investigations. As the closest phylogenetic relatives to humans, nonhuman primates (NHPs) play an indispensable role in biomedical research and can serve as a critical translational bridge between basic studies performed in rodent models and clinical studies in humans [4]. In our previous work, several rhesus monkeys with spontaneous type 2 diabetes mellitus (T2DM) were screened and found within our colony, and their T2DM-related parameters were similar to those of humans [5]. We previously demonstrated cardiomyopathy in spontaneous T2DM rhesus monkeys. The changes in conventional echocardiography and cine magnetic resonance imaging (MRI) in spontaneous T2DM rhesus monkeys are similar to those found in humans with T2DM. These findings indicate an early stage of DCM characterized by diastolic dysfunction with preserved left ventricular ejection fraction (LVEF) [6]. Therefore, these spontaneous T2DM rhesus monkeys can be useful for preclinical and translational investigations in T2DM [7].

DCM is usually asymptomatic in the early stages of its evolution [8]. Early detection and intervention of asymptomatic DCM could improve the quality of life of patients and reduce both morbidity and mortality [9]. Imaging techniques, particularly cardiac MR (CMR), are the mainstay methods to recognize asymptomatic metabolic cardiomyopathy, as well as to further monitor pathological alterations and potential responses to therapy. Myocardial deformation measurements that track intramyocardial features detected between the epicardial and endocardial myocardial tissue boundaries have emerged as superior parameters of LV function and performance by reflecting both systolic and diastolic LV functions in the early stage of DCM [10, 11]. CMR tagging is considered the gold standard for measuring regional myocardial strain; it was developed to successfully provide a comprehensive characterization of rhesus monkey cardiac function in our previous study [12]. Healthy rhesus monkeys also have similar deformation characteristics to humans. However, the tagging approach has limited clinical applicability because it requires additional tagging images and prolonged imaging times.

CMR tissue tracking (CMR-TT) is a method for the noninvasive assessment of myocardial deformation applied to routine cine CMR acquisitions, with no additional image acquisition and no increase in scan time [13]. This technique shows sufficient agreement with CMR tagging [14], as well as higher reproducibility and lower observer variability than speckle-tracking echocardiography [15]. Recently, three-dimensional (3D) CMR-TT has been developed using long-axis (LA) and short-axis (SA) cine images [16]. Moreover, 3D analysis could theoretically reduce artifacts in deformation such as those that may result from through-plane displacements of 3D structures [17]. However, the performance of two-dimensional (2D)/3D CMR-TT in the early stage of DCM is unknown.

In this study, we aimed to investigate the early cardiac function changes and myocardial deformation characteristics by using 2D/3D CMR-TT in a spontaneous T2DM rhesus monkey model with early-stage DCM, compared with CMR tagging. This study was expected to confirm the value of spontaneous T2DM rhesus monkeys in research on diabetic cardiomyopathy and establish an effective noninvasive CMR method to evaluate cardiac function.

## Methods

### Animals

Ten spontaneous T2DM rhesus monkeys with isolated diastolic dysfunction (DD) and 9 age-matched nondiabetic (ND) normal monkeys screened from 300 rhesus monkeys were recruited in this study. The animals had ad libitum access to a standard monkey diet (calories provided from protein, 17%; from fat, 30%; and from carbohydrates, 53%). Methods for determination of fasting plasma glucose (FPG) and other blood biochemical indicators were employed as previously described [5]. The inclusion criteria used for the T2DM rhesus monkey selection [5, 6, 18] and the ultrasonography diagnostic criteria for diastolic dysfunction were determined based on a previous study. The main indicators were a peak velocity of the mitral annulus during rapid ventricular filling (e, cm/s) < 8 and a ratio of early transmittal velocity to tissue Doppler mitral annular early diastolic velocity (e/e’) > 10 [19]. The exclusion criteria for systolic dysfunction were an ejection fraction (EF) < 65% and shortening fraction (FS) < 35% [20]. Meanwhile, both T2DM-DD and ND monkeys met the criteria of systolic blood pressure ≤ 140 mmHg and diastolic blood pressure ≤ 80 mmHg to exclude hypertension.

### Animal experiments

Gravimetry and metabolic profiles of all enrolled monkeys were obtained during a 4-week acclimation period. FPG levels were determined semimonthly 2 times. Total cholesterol (TC), triglycerides (TG), low-density lipoprotein cholesterol (LDL-c), high-density lipoprotein cholesterol (HDL-c), and other metabolic profiles were determined at the end of the acclimation period. Body weight was measured after the last blood collection. Echocardiography was performed on all monkeys within 48 h after the last final blood collection. MRI was conducted a week after echocardiography. All monkeys fasted approximately 12 h prior to image examination. Blood pressure was measured immediately before each image examination. Animals were sedated with ketamine hydrochloride (100 mg/mL, Bioniche Teoranta, Inverin Co, Galway, Ireland) at a dosage of 10 mg/kg intramuscularly immediately before the echocardiography imaging session. The animals were anesthetized with ketamine hydrochloride (10 mg/kg, given intramuscularly) and propofol (4 to 10 mg/kg, given intravenously; United States Pharmacopeia grade 100%; RWD Life Science, San Diego, CA, USA), followed by tracheal intubation with artificial ventilation to control respiration (tidal volume, 150 to 200 mL/min; respiratory rate, 15–20 breaths/min; isoflurane dosage, 0.2 to 0.3 mL/kg) before and during each CMR imaging session. The breath-holds were enforced by taking the animal temporarily off the ventilator for an average of 30–35 s. The CMR imaging protocol was performed twice (test and retest scans) in 9 ND animals 1 week apart by two independent scanners. The animal was pulled completely out of the scanner, the coils were repositioned, and the animal was re-landmarked to the magnet isocenter prior to the retest scan. At the end of the experiment, all rhesus monkeys were kept alive.

### Cardiovascular magnetic resonance imaging

MR studies were performed with the monkeys in the supine position using a 3.0-T clinical MRI system (MAGNETOM Trio, Siemens Medical Solutions, Erlangen, Germany) with a 32-channel cardiac surface coil (Siemens). Sterile drapes were used to separate the monkeys from the examination bed, and a dedicated quilt was used to keep the monkeys warm during scanning.

CMR cine sequences were performed to obtain two-chamber, four-chamber and short-axis (SA) views that included the entirety of both ventricles (10–14 slices) via a steady-state free precession with retrospective electrocardiogram triggering. The cine steady-state free precession sequence parameters were as follows: echo time/repetition time = 1.41 ms/26.48 ms; field of view = 160 × 160 mm; number of excitations = 2; matrix = 256 × 256; flip angle = 50°; slice thickness = 5.0 mm; slice gap = 2 mm; bandwidth = 888 Hz/Px; and 25 phases per cardiac cycle.

Three SA images selected at the LV basal (mitral valve), middle (papillary muscle), and apical levels, as well as long-axis (LA) images in two- and four-chamber views selected in advance were acquired for tagging images using a gated, multiphase, segmented gradient echo pulse sequence with a 1–2-1 spatial modulation of the magnetization tagging preparation sequence [21, 22]. Two sets of tagging datasets with orthogonal in-plane tagging modulations were acquired for each of the three SA slices and the two- and four-chamber LA slices. The selected imaging parameters were as follows: field of view = 200 × 200 mm; slice thickness = 5 mm; flip angle = 8°; imaging pixel matrix = 208 × 208; segments = 4; echo spacing = 6 ms; repetition time/echo time = 23.84 ms/2.82 ms; bandwidth = 511 Hz/Px; tag separation = 5 mm; and temporal resolution = 12.72 ms. No parallel imaging was used. All tagging data were obtained during ventilator-induced breath-holds, which were an average of 35 s long, to eliminate breathing-related motion artifacts.

### Image analysis

All tagged imaging datasets were analyzed using harmonic phase methodology [23, 24] in dedicated software, which was previously developed and modified in-house in the MATLAB environment (Math Works, Natick, MA, USA), as we described previously [12]. For each SA slice, a user-defined mesh with three layers (endocardium, mid-wall and epicardium) and four (apical) to six (mid and basal) segments was superimposed on the myocardium at the first timeframe. For each LA slice, the 2D displacement path lines of all voxels in the myocardium were tracked. Average regional circumferential strain was computed from the SA-tagged slices, and regional longitudinal strain was computed from the LA slices. Regions defined on the SA and LA slices were in accordance with the American Heart Association standardized cardiac segmentation guidelines [25]. Numerical data were outputted for further postprocessing to quantify peak systolic circumferential strain (Ecc, %), time to peak systolic circumferential strain (tEcc, s), peak diastolic circumferential strain rate (CSR, %/s), peak systolic longitudinal strain (Ell, %), time to peak systolic longitudinal strain (tEll, s) and peak diastolic longitudinal strain rate (LSR, %/s) for LV global.

CMR-TT analyses were performed using dedicated software (cvi^42^, Circle Cardiovascular Imaging Inc., Calgary, Alberta, Canada), a commercially available product commonly used to analyze CMR images. SA cine CMR images and the corresponding LV two- and four-chamber LA images with cross-referencing locations were uploaded into the software, which reconstructs 2D and 3D models that are used for analyses of 2D and 3D circumferential and longitudinal deformation parameters. In this model, the most apical section showing the LV cavity at end-systole was considered the 0% LV location. The most basal section, including the complete circumference of the myocardium at end-systole, was considered the 100% LV location. The endocardial and epicardial borders on the end-diastolic frame of each slice were manually delineated and corrected and automatically propagated; they were visually assessed and selected at the largest phase (end-diastolic). A 2D incompressible deformable model of the myocardium with individual image slices (e.g., LA or SA acquisitions) over the cardiac cycle and a 3D deformable model of the myocardium in between the endo and epi surfaces generated by interpolating the tracked boundaries from the 2D algorithm were both used to obtain 2D and 3D deformation quantities. Ecc (%), tEcc (s), CSR (%/s), Ell (%), tEll (s) and LSR (%/s) were derived in both 2D and 3D.

### Statistical analysis

All global deformation biomarkers obtained using the tagging and CMR-TT methods were compared by paired-samples t-test, intraclass correlation coefficient (ICC) and Bland-Altman analysis [26] after the data were tested for normal distribution. The test-retest repeatability was assessed using the ICC, and Bland-Altman analysis was used to quantify test-retest reliability. ICC estimates and their 95% confidence intervals were calculated based on a single-measurement, absolute-agreement, 2-way mixed-effects model. The parameters defined above were compared between the T2DM-DD and ND groups using the independent-samples t-test. The correlations were evaluated using Pearson’s correlation coefficient (r). Statistical tests were performed with SPSS software version 22.0 (SPSS Inc., Chicago, IL, USA), GraphPad software version 6.00 (GraphPad Software, La Jolla, California, USA) and Medcalc software version 15.2.2 (MedCalc Software bvba, Ostend, West Flanders, Belgium). A *p* value < 0.05 was considered statistically significant.

## Results

### Characteristics and metabolic profile

Complete data were available for all 19 monkeys in our study (10 T2DM-DD, 9 ND; Table 1). No difference was found with regard to age, body mass index or blood pressure. T2DM-DD monkeys had higher FPG values (4.71 ± 0.66 vs 4.10 ± 0.38 mmol/L, *p* < 0.05). Higher HDL-c (2.29 ± 0.69 vs 1.53 ± 0.32 mmol/L, *p* < 0.01) levels were observed in the T2DM-DD group. No difference was found with regard to age (13.10 ± 1.52 vs 12.22 ± 1.72 cm/s, *p* < 0.01). However, e/e’ was found to be significantly higher in the T2DM-DD group than in the ND group (13.47 ± 2.80 vs 8.07 ± 1.41, *p* < 0.01). No significant difference was noted in the conventional parameters of systolic function, including LVEF and FS (75.38 ± 6.65 vs 75.28 ± 2.75%, *p* > 0.05 for LVEF; 42.93 ± 6.60 vs 42.25 ± 2.51%, *p* > 0.05 for FS).

**Table 1:** Comparison of clinicopathological characteristics between groups of rhesus monkeys

Variable	T2DM-DD monkeys (*n* = 10)	ND monkeys (*n* = 9)	*p* value
Age, years	13.10 ± 1.52	12.22 ± 1.72	0.25
BMI, kg/m^2^	31.00 ± 10.26	26.20 ± 7.04	0.26
Systolic blood pressure, mm Hg	136.57 ± 19.03	119.67 ± 14.36	0.07
Diastolic blood pressure, mm Hg	65.40 ± 7.56	64.38 ± 14.38	0.85
Echocardiography			
EF, %	75.38 ± 6.65	75.28 ± 2.75	0.97
FS, %	42.93 ± 6.60	42.25 ± 2.51	0.78
e (cm/s)	6.05 ± 1.41	10.74 ± 1.49	0.00**
e/e’	13.47 ± 2.80	8.07 ± 1.41	0.00**
Diabetic status			
FPG, mmol/L	4.71 ± 0.66	4.10 ± 0.38	0.03*
Lipid status			
TC, mmol/L	3.24 ± 0.67	2.76 ± 0.50	0.10
LDL, mmol/L	1.24 ± 0.23	1.42 ± 0.51	0.33
HDL, mmol/L	2.29 ± 0.69	1.53 ± 0.32	0.01**
TG, mmol/L	0.42 ± 0.12	0.40 ± 0.16	0.81

Results are expressed as the means±SD

**p < 0.01, *p < 0.05

### Global deformation analysis of DCM

**Fig. 1:**
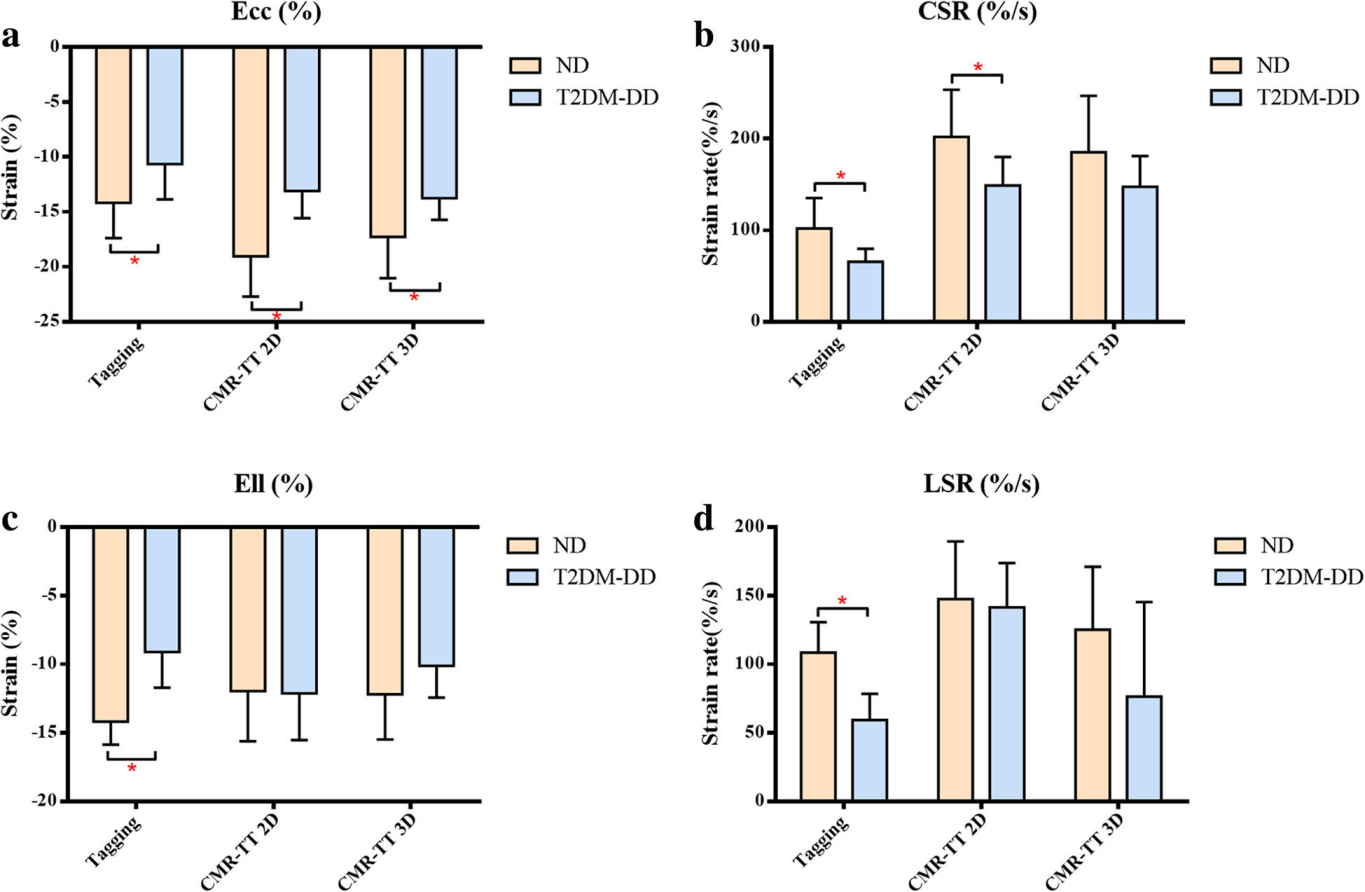
Column graphs of deformations in the two groups. Peak systolic circumferential strain, Ecc (**a**); Peak diastolic circumferential strain rate, CSR (**b**); Peak systolic longitudinal strain, Ell (**c**); Peak diastolic longitudinal strain rate, LSR (**d**).*p < 0.05 vs. ND group. Data are expressed as the means ± SD

A summary of the comparison of T2DM-DD monkeys versus ND monkeys for all deformation parameters from 2D/3D CMR-TT and tagging is presented in Figure 1. Radial strain was not adopted due to its large ranges between studies and segmental strain variability. Global values were adopted because these are more robust and reproducible than regional values and have been more widely applied for diseases associated with diffuse and homogeneous abnormalities. The absolute value represents strain amplitude and strain rate magnitude. The symbol represents the direction of strain and strain rate.

### Tagging

**Fig. 2:**
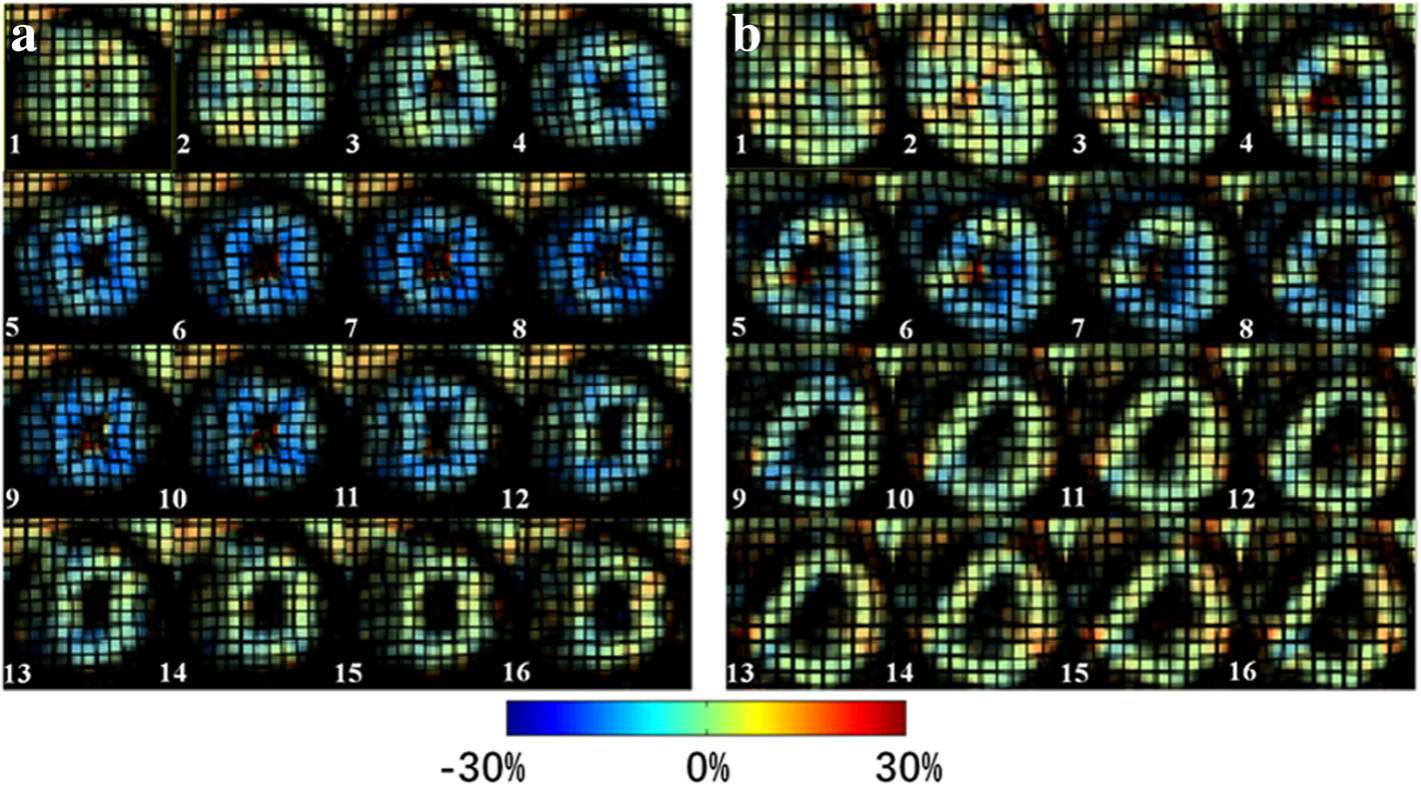
Representative CMR tagging circumferential strain color maps evolution over a cardiac cycle. Circumferential strain color maps in a T2DM-DD monkey (**b**) and a ND monkey (**a**). Circumferential strain color maps with tag overlay in a mid-ventricular short-axis slice over the entire cardiac cycle depicting systolic compression and diastolic relaxation. Systolic compression and diastolic relaxation were impaired in the T2DM-DD monkeys

CMR tagging imaging revealed that both systolic and diastolic deformation functions were impaired in T2DM-DD monkeys. The related parameters were all statistically significant in the ND group. During diastole, the absolute values of CSR and LSR were much lower in the T2DM-DD group than in the ND group (65.50 ± 14.48 vs 65.50 ± 14.48%/s, *p* < 0.01 for CSR; 59.43 ± 19.17 vs 108.46 ± 22.33%/s, *p* < 0.01 for LSR). Meanwhile, the absolute values of Ecc and Ell were lower in the T2DM-DD group than in the ND group during systole (− 10.63 ± 3.23 vs − 14.18 ± 3.19%, *p* < 0.05 for Ecc; − 9.11 ± 2.59 vs − 14.17 ± 1.68%, *p* < 0.01 for Ell). This finding indicates that the cardiac function in both cardiac phases was impaired (Figure 2). No significant difference was found in the time to peak strain between the two groups in this study (0.26 ± 0.05 vs 0.25 ± 0.04 s, *p* > 0.05 for tEcc; 0.26 ± 0.04 vs 0.24 ± 0.05 s, *p* > 0.05 for tEll).

### CMR-TT

**Fig. 3:**
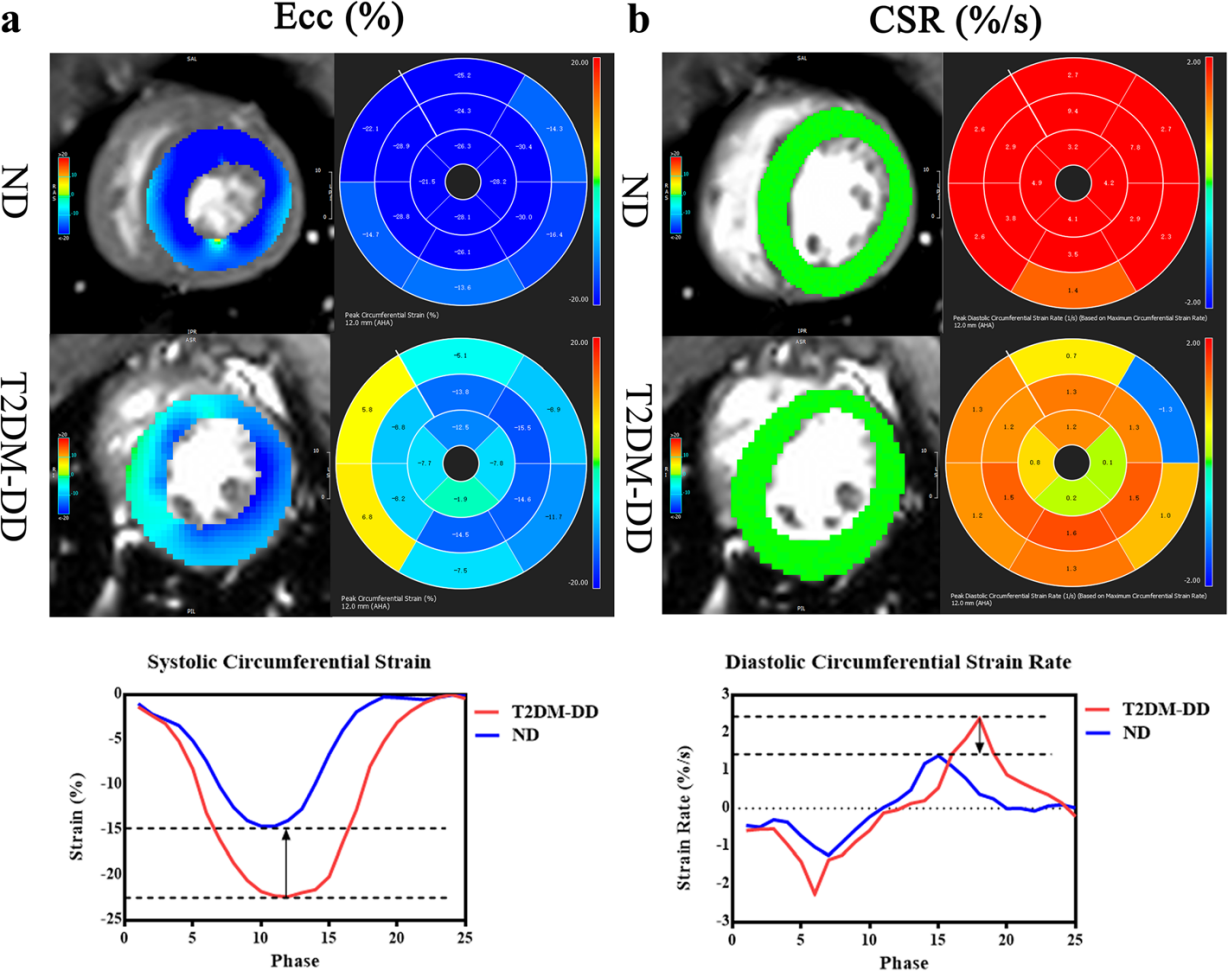
Representative 2D CMR-TT-derived Ecc and CSR in the two groups. The colored tissue-tracking 2D maps, 16-segment models and strain-time curves in a cardiac cycle of Ecc showed that the systolic function was impaired in the T2DM-DD monkey (**a**). The colored tissue-tracking 2D maps, 16-segment models and strain-time curves in a cardiac cycle of CSR showed that the diastolic function was impaired in the T2DM-DD monkeys (**b**)

Measures of 2D CMR-TT showed significant reductions of Ecc and CSR (absolute value) in T2DM-DD monkeys compared with ND monkeys (− 13.10 ± 2.47 vs − 19.03 ± 3.69%, *p* < 0.01 for Ecc; 148.90 ± 31.27 vs 202.00 ± 51.88%/s, *p* < 0.01 for CSR) (Figure 3). Only the absolute value of global Ecc decreased significantly in T2DM-DD monkeys in 3D CMR-TT (− 13.77 ± 1.98 vs − 17.26 ± 3.78%, *p* < 0.05).

### Agreement between 2D/3D CMR-TT and CMR tagging

**Fig. 4:**
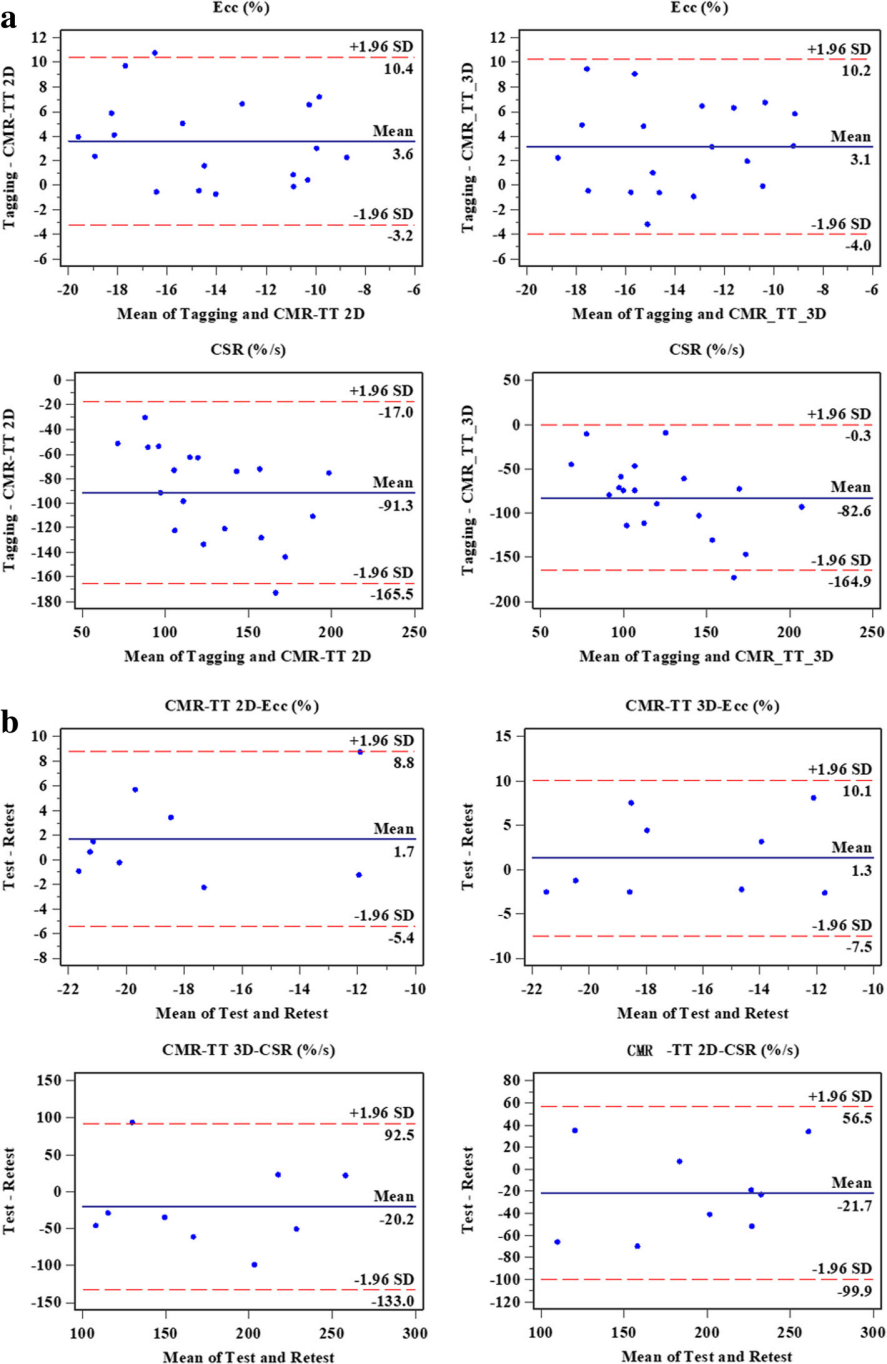
Plots depicting Bland-Altman repeatability (**a**) and consistency (**b**) analysis for 2D/3D CMR-TT-derived circumferential deformation parameters. The x-axis represents the mean, and the y-axis represents the difference. The central horizontal line indicates the mean value of the difference. The solid black lines at the two extremes represent the means ± 1.96 standard deviations of the difference

The absolute values of CMR-TT-derived Ecc (tagging vs CMR-TT 2D/3D: -12.31 ± 3.61 vs − 15.91 ± 4.28/ -15.42 ± 3.40%, *p* < 0.01) and CSR (tagging vs CMR-TT 2D/3D: 82.80 ± 30.73 vs 174.05 ± 49.27/165.37 ± 51.36%/s, *p *< 0.01) in both the T2DM-DD and ND groups were higher than the corresponding tagging values. Both CMR-TT 2D- and 3D-derived Ecc and CSR correlated with the corresponding tagging values, with statistically significant ICC values (Ecc: 0.61 for 2D, 0.51 for 3D; CSR: 0.29 for 2D, 0.30 for 3D, *p* < 0.05) and a relatively narrow limit of agreement and a concentrated distribution in the Bland-Altman analysis (Figure 4). CMR-TT 2D, 3D-derived tEcc (tagging vs CMR-TT 2D/3D: 0.26 ± 0.05 vs 0.23 ± 0.03/0.23 ± 0.03, *p* < 0.05) and CMR-TT 2D-derived tEll (0.25 ± 0.04 vs 0.23 ± 0.05, *p* < 0.01) were higher than the corresponding tagging values. CMR-TT 3D-derived tEll correlated with the corresponding tagging value, with a statistically significant ICC (0.75, *p* < 0.05). There were no significant correlations in the other deformation parameters.

### Test-retest reproducibility of 2D/3D CMR-TT and tagging

Results obtained from test-retest reprocibility analysis are shown in Table 2 for all the systolic and diastolic deformation parameters. The test-retest reproducibility of CMR-TT 2D-derived Ecc and CSR (0.77 for Ecc, 0.86 for CSR, *p* < 0.01) was better than that of the 3D-derived values (Figure 4b) but poorer than that of those derived from tagging. In addition, the ICC values were acceptable for CMR-TT 2D-derived Ell and LSR (0.90 for Ell, 0.87 for LSR, *p* < 0.01), which were better than those obtained from the 3D-derived values and tagging. For 3D CMR-TT, the test-retest repeatability was unacceptable for Ecc, CSR, tEll and LSR (*p* > 0.05). CMR-TT 3D-derived tEcc and Ell showed acceptable reproducibility (0.84 for tEcc, 0.77 for Ell, *p* < 0.05), which were not better than the values obtained from tagging.

**Table 2:** Test-retest reproducibility analysis of tagging and 2D/3D CMR-TT

Total study group (*n* = 19)	Intraclass correlation coefficient (95% CI)	Bland-Altman analysis	
		Mean bias (%)	LOA
Tagging			
Ecc (%)	0.83** (0.32/0.96)	1.30	−3.90/6.40
tEcc (s)	0.96** (0.82/0.99)	0.01	−0.03/0.05
CSR (%/s)	0.92** (0.64/0.98)	−4.30	−48.70/40.00
Ell (%)	0.73* (−0.03/0.94)	1.10	−2.10/4.40
tEll (s)	0.93** (0.71/0.98)	0.01	−0.04/0.05
LSR (%/s)	0.66*(−0.20/0.92)	−13.60	−61.10/33.80
2D CMR-TT			
Ecc (%)	0.77* (−0.01/0.95)	1.70	−5.40/8.80
tEcc (s)	0.53 (−1.58/0.90)	0.00	−0.06/0.06
CSR (%/s)	0.86** (0.36/0.97)	−21.70	−99.90/56.50
Ell (%)	0.90** (0.55/0.98)	1.60	−2.50/5.60
tEll (s)	0.31 (−2.07/0.84)	0.04	−0.12/0.19
LSR (%/s)	0.87** (0.43/0.97)	−31.10	−88.60/26.40
3D CMR-TT			
Ecc (%)	0.62 (−0.66/0.91)	1.30	−7.50/10.10
tEcc (s)	0.84** (0.27/0.97)	0.00	−0.04/0.05
CSR (%/s)	0.71 (−0.18/0.93)	−20.20	− 133.00/92.50
Ell (%)	0.77* (0.05/0.95)	0.90	−4.50/6.30
tEll (s)	−0.28 (−3.43/0.69)	− 0.02	−0.08/0.05
LSR (%/s)	0.26 (−1.52/0.83)	−22.90	− 118.6/72.80

**p < 0.01, *p < 0.05

## Discussion

In this study, LV systolic and diastolic deformation functions were impaired, as demonstrated by tagging and CMR-TT in spontaneous T2DM-DD monkeys, similar to the impairment found in diabetic patients. 2D CMR-TT-derived Ecc and CSR were accurate and robust biomarkers used to evaluate the myocardial systolic and diastolic functions of the spontaneous T2DM rhesus monkeys.

From the present mechanistic point of view, DCM seems to progress through an initial asymptomatic period through diastolic heart failure with normal EF, followed by systolic dysfunction accompanied by heart failure with reduced EF [3]. In our previous echocardiography study, diastolic dysfunction was the earliest functional alteration in DCM, which led to contractile abnormality in spontaneous T2DM rhesus monkeys [6]. Consistent with previous conclusions [11], T2DM monkeys with diastolic dysfunction but preserved EF (T2DM-DD) by echocardiography were selected as the research objects of early DCM in this study. Analysis of myocardial deformation of those T2DM-DD monkeys showed that tagging-derived systolic and diastolic deformation parameters were all impaired, particularly LV Ecc, Ell, CSR and LSR, with high test-retest reproducibility. This result is supported by speckle-tracking echocardiography, which demonstrated systolic tissue dysfunction, measured as a decrease in LV longitudinal strain, in patients who were assumed to have “isolated” diastolic dysfunction [27–30]. These findings indicate that systolic and diastolic dysfunctions occur concomitantly with a range of severity in the progression to DCM, and LVEF cannot be effectively used to detect abnormal systolic function in the early stage of DCM. A recent study reported that overweight and obesity are associated with impaired LV systolic function in both the T2DM and non-T2DM populations [31], and metabolic abnormalities may facilitate this process [32]. These results indicate that weight and obesity, both independent risk factors for T2DM, may induce subclinical damage to myocardial systolic function in the early stage of metabolic abnormality in diabetes.

In the tagging study by Fonseca et al., the absolute values of systolic Ecc, Ell, and diastolic CSR and LSR were lower in patients with T2DM, diastolic dysfunction, and a normal EF [11], who exhibit similar deformation characteristics to those of our T2DM monkeys. This consistency between monkeys and humans may be related to the similarities in the metabolism and pathophysiology of diabetes [33, 34] and close myocardial fiber direction and structure [35]. However, Levelt et al. reported that only mid-ventricular systolic circumferential and global longitudinal strains were impaired in patients with T2DM [36]. They analyzed the pEcc, Ell, CSR and LSR data from a single slice in the mid-short-axis and horizontal LA views only, which may lead to insufficient parameter stability due to limited raw data. In this study, the spontaneous T2DM rhesus monkey cardiac deformation characteristics closely resembled those of a T2DM patient’s heart, which could be effective for the investigation and preclinical testing of novel T2DM therapeutic agents, with a high potential for translatability to humans.

To date, few studies have reported whether CMR-TT can be used as an easier, effective, consistent evaluation technique for DCM. In this report, we evaluated the effectiveness of CMR-TT in DCM and its consistency with tagging. The results show that there was significant injury in 2D CMR-TT-derived Ecc and CSR in T2DM-DD monkeys. All 2D CMR-TT-derived circumferential and longitudinal strains and strain rates showed good test-retest reproducibility, which is in accordance with previously published literature [37]. Compared with longitudinal deformation parameters, the 2D CMR-TT-derived circumferential strain and strain rate were higher, acceptably consistent with CMR tagging, in accordance with previous reports [37–39]. Overall, 2D CMR-TT-derived Ecc and CSR are powerful biomarkers for distinguishing the myocardial systolic and diastolic functions of spontaneous T2DM monkeys from those of non-T2DM monkeys, with high test-retest reproducibility and acceptable agreement with tagging.

The technology of tissue tracking refers to methods of identifying a peculiar pattern of a set of control points. In the 2D algorithm, the deformation of the model is assumed to be completely determined by a set of control points placed on the middle curve of the myocardial wall to individual image slices (e.g., long- or short-axis acquisitions). In the 3D algorithm, the deformation of the model is determined by a set of control points generated by interpolating the tracked boundaries from the 2D algorithm using both long- and short-axis image information. In this study, we found that the 2D algorithm had higher reproducibility than the 3D algorithm. Furthermore, 3D CMR-TT could only detect systolic dysfunction in T2DM-DD monkeys. In comparison, Liu et al. considered that 3D CMR-TT offers superior reproducibility compared with 2D CMR-TT [40]. The cause of the difference may be attributed to their analyses of 2D strain at the mid-left ventricle in the SA view and 3D strain in all SA and LA slices. In the present study, 2D and 3D deformations were analyzed at all SA and LA slices. In addition, they only acquired 3D values in a normal population. Therefore, it is difficult to determine whether 3D CMR-TT would provide incremental value in disease cohorts. There is evidence from previous studies that the problem of through-plane motion can be solved using 3D techniques compared with 2D algorithms [41, 42]. Nevertheless, 3D images present a substantially (at least 3–4×) lower spatial and temporal resolution than their 2D counterpart. This finding may be related to the fact that experience with the 3D algorithm is still limited. We infer that these findings may be the reason why 3D results are less stable and effective. Therefore, further theoretical and empirical studies are required to confirm these findings.

## Conclusions

To the best of our knowledge, this is the first study to evaluate myocardial deformation in spontaneous T2DM rhesus monkeys with the CMR-TT analysis method. We have concluded that early cardiac function and myocardial deformation characteristics of spontaneous T2DM rhesus monkeys are similar to those found in human T2DM. Meanwhile, CMR-TT may be used as an integral component of CMR to more easily evaluate cardiac function in DCM. 2D CMR-TT-derived Ecc and CSR have proven application value due to their ability to detect early deformation changes, their test-retest reliability, and their correlation to tagging in the onset stage of DCM. Because we used a rare nonhuman primate model of T2DM, the current study included a small cohort (*n* = 9 for the T2DM-DD group and *n* = 10 for the ND group), which could have contributed to the lower reproducibility and reliability in the quantified biomarkers. Therefore, a larger sample size is required in future studies.

## Abbreviations

BUN: Blood urea nitrogen
CMR: Cardiac magnetic resonance imaging
CMR-TT: CMR tissue tracking
CSR: Peak diastolic circumferential strain rate
DCM: Diabetic cardiomyopathy
e: Peak velocity of mitral blood flow during the rapid ventricular filling
e/e’: The ratio of early transmittal velocity to tissue Doppler mitral annular early diastolic velocity
e’: Peak velocity of mitral annulus during the rapid ventricular filling
Ecc: Peak systolic circumferential strain
Ell: Peak systolic longitudinal strain
FPG: Fasting plasma glucose
FS: Shortening fraction
ICC: Intraclass correlation coefficient
LSR: Peak diastolic longitudinal strain rate
T2DM: Type 2 diabetes mellitus
TC: Total cholesterol
tEcc: Time to peak systolic circumferential strain
tEll: Time to peak systolic longitudinal strain
TG: Triglycerides
LVEF: Left ventricular ejection fraction
